# Educational inequalities in self-rated health across US states and European countries

**DOI:** 10.1007/s00038-017-0981-6

**Published:** 2017-05-22

**Authors:** Patrick Präg, S. V. Subramanian

**Affiliations:** 1 Department of Sociology and Nuffield College, University of Oxford, Oxford, UK; 2 Department of Social and Behavioral Sciences, Harvard Chan School of Public Health, Boston, MA US

**Keywords:** International comparison, Self-rated health, Health inequalities, Spatial inequalities

## Abstract

**Objectives:**

The US shows a distinct health disadvantage when compared to other high-income nations. A potential lever to reduce this disadvantage is to improve the health situation of lower socioeconomic groups. Our objective is to explore how the considerable within-US variation in health inequalities compares to the health inequalities across other Western countries.

**Methods:**

Representative survey data from 44 European countries and the US federal states were obtained from the fourth wave of the European Values Study (EVS) and the 2008 wave of the Behavioral Risk Factor Surveillance System. Using binary logistic regression, we analyze different forms of educational inequalities in self-rated health (SRH), adjusted for age and sex.

**Results:**

The extent of educational inequalities in SRH varies considerably over European countries and US states; with US states in general showing greater inequality, however, differences between US states and European countries are less clear than commonly assumed.

**Conclusions:**

The US has considerable differences in educational inequalities in SRH across geographic locations. To understand the reasons for the US health disadvantage, comparative research has to take into account the vast variation in health inequalities within the US.

## Introduction

When compared to other industrialized, high-income countries, the US population exhibits a distinct health disadvantage across important health outcomes, such as infant mortality, heart disease, sexually transmitted diseases, injuries from violence and motor vehicle crashes, and life expectancy (Avendano and Kawachi 2014; Bezruchka 2012; Crimmins et al. 2011; Wang et al. 2016). While differences in health care might be one cause of this disadvantage, it is unlikely that they are the main driver (Avendano and Kawachi 2014): the US has the highest health care spending in the world (Dieleman et al. 2016). Furthermore, the wide range of health indicators where the US is at a disadvantage underlines that medical care deficiencies cannot be the sole cause. A potential lever suggested to eliminate the overall health disadvantage of the US population is to improve the health situation of lower socioeconomic groups (Adler et al. 2016; Avendano and Kawachi 2014; Schroeder 2016).

Various studies (Avendano et al. 2009; Banks et al. 2006, 2016; Martinson and Reichman 2016; McDonough et al. 2010; Semyonov et al. 2013; Van Hedel et al. 2016; Woolf and Aron 2013) have shown that the US as a whole indeed exhibits greater health inequalities by socioeconomic status than European countries. In addition, population health and health inequalities in the US vary greatly by geographical location (Chetty et al. 2016; Currie and Schwandt 2016; Ezzati et al. 2008; Murray et al. 2006) and this geographical variation is not explainable by race of the population or health care access and utilization alone (Avendano and Kawachi 2014; Murray et al. 2006). We define health inequalities in this context as educational inequalities in self-rated health (SRH). While there are other dimensions to health inequalities such as sex and race, we believe that socioeconomic status is a particularly salient dimension, and that education is a particularly good indicator for socioeconomic status.

In this study, we explore how within-US variation in the education–SRH association compares to the variation between European countries. Some of the association between education and SRH is due to biological factors such as genes, and these biological factors are commonly assumed not to vary across spatial contexts. Thus, variation in the size of the education–SRH association across different regional and national contexts points to the importance of social factors in the emergence of health inequalities and illustrates the potential malleability of health inequalities by social factors such as policy interventions. With Europe and the US, we contrast two particularly interesting cases: compared to Europe with its diverse and distinct historical trajectories and its resulting institutional variety (Mackenbach et al. 2013), the US has experienced a rather homogeneous development in terms of institutions; however, the US still exhibits a well-known within-country heterogeneity in terms of health.

We are investigating educational inequalities in SRH in 44 European countries and the 50 federal states of the US. We examine four key indicators of population health and health inequalities; next to presenting the overall prevalence of poor health, we show the prevalence of poor health among the lower educated, an oft-overlooked indicator of health inequalities. In line with other recent research on health inequalities (Dudal and Bracke 2016; Kulhánová et al. 2014; Vandenheede et al. 2015), we also rely on both absolute and relative measures of educational inequalities in SRH.

Our study makes contributions to two contemporary debates in public health—the debate as to how health inequalities differ between the US and similar rich countries (e.g., Martinson and Reichman 2016; Van Hedel et al. 2016) and the debate about within-US differences in health inequalities (e.g., Chetty et al. 2016). By virtue of doing so, we extend the ‘methodological nationalism’ (Wimmer and Glick Schiller 2002) prevalent in the majority of current comparative research on health inequalities. Researchers often rely on the nation-state as a seemingly ‘natural’ unit of analysis (Olafsdottir et al. 2013), but the focus on nation states as units of analyses can mask important heterogeneities within countries. Breaking down the US into its 50 federal states and comparing them to European countries is a first step toward looking into the black box of a nation-state in cross-national research, exploring the variance of health inequalities within and between countries.

## Methods

We combine the 2008 waves of the European Values Study (EVS) and the Behavioral Risk Factor Surveillance System (BRFSS). The 2008 wave of the EVS (2011) provides health self-ratings for samples of individuals from all European countries with 100,000 or more inhabitants. Sampling and questionnaire translation procedures followed high quality standards to ensure comparability between countries. Our European sample includes health information from 44 European countries and the region of Kosovo. The BRFSS (CDC 2008) is a US-state representative survey of the adult population living in households. The BRFSS comprises large samples from the 50 federal states as well as the District of Columbia. We chose the 2008 wave of the BRFSS for our analyses due to the temporal proximity to the EVS and removed the US territories Guam, Virgin Islands, and Puerto Rico from the analysis.

For our analyses, we restricted the age range from 25 to 75 years to ensure that respondents’ educational attainment process is completed and to remove health variation attributable to differential life expectancies across areas. We restricted the BRFSS to non-Hispanic Whites to focus on variation not attributable to racial health inequalities in the US. This is common practice in research comparing health inequalities between the US and European countries (Avendano et al. 2009; Banks et al. 2006). The number of respondents in each European country is about *N* = 1100 and in each US state around *N* = 5400, which allows us to meaningfully compare differences between the federal states and European countries. The comparatively small sample sizes in the EVS do not allow us to break down European countries into subnational units. Exact sample sizes per country and federal state as well as name abbreviations are reported in Table 1.

**Table 1:** Sample sizes and country/state codes, 2008

Abbreviation	Country	*N*	Abbreviation	Federal state	*N*
AL	Albania	1172	US-AK	Alaska	1643
AM	Armenia	1151	US-AL	Alabama	3756
AT	Austria	1223	US-AR	Arkansas	3693
AZ	Azerbaijan	1137	US-AZ	Arizona	3370
BA	Bosnia Herzegovina	1217	US-CA	California	5950
BE	Belgium	1232	US-CO	Colorado	8334
BG	Bulgaria	1241	US-CT	Connecticut	3868
BY	Belarus	1155	US-DC	District of Columbia	1697
CH	Switzerland	1058	US-DE	Delaware	2699
CY	Cyprus	1159	US-FL	Florida	7050
CZ	Czech Republic	1459	US-GA	Georgia	3379
DE	Germany	1771	US-HI	Hawaii	1900
DK	Denmark	1278	US-IA	Iowa	4655
EE	Estonia	1232	US-ID	Idaho	3942
ES	Spain	1166	US-IL	Illinois	3318
Europe	(Entire EVS data set)	54,066	US-IN	Indiana	3330
FI	Finland	1000	US-KS	Kansas	6296
FR	France	1224	US-KY	Kentucky	6442
GE	Georgia	1232	US-LA	Louisiana	3798
GR	Greece	1209	US-MA	Massachusetts	13,388
HR	Croatia	1130	US-MD	Maryland	5902
HU	Hungary	1198	US-ME	Maine	5435
IE	Ireland	781	US-MI	Michigan	5963
IS	Iceland	646	US-MN	Minnesota	3368
IT	Italy	1203	US-MO	Missouri	3641
LT	Lithuania	1190	US-MS	Mississippi	4369
LU	Luxembourg	1115	US-MT	Montana	4977
LV	Latvia	1184	US-NC	North Carolina	10,099
MD	Moldova	1188	US-ND	North Dakota	3885
ME	Montenegro	1232	US-NE	Nebraska	12,191
MK	Macedonia	1242	US-NH	New Hampshire	5404
MT	Malta	1222	US-NJ	New Jersey	6898
NL	Netherlands	1260	US-NM	New Mexico	2920
NO	Norway	916	US-NV	Nevada	3014
PL	Poland	1183	US-NY	New York	4987
PT	Portugal	1252	US-OH	Ohio	9069
RO	Romania	1207	US-OK	Oklahoma	4930
RS	Serbia	1241	US-OR	Oregon	3547
RU	Russian Federation	1166	US-PA	Pennsylvania	8974
SE	Sweden	1046	US-RI	Rhode Island	3286
SI	Slovenia	1098	US-SC	South Carolina	5529
SK	Slovak Republic	1263	US-SD	South Dakota	4884
TR	Turkey	1928	US-TN	Tennessee	3651
UA	Ukraine	1222	US-TX	Texas	5518
UK	United Kingdom	1527	US-UT	Utah	4075
XK	Kosovo	1110	US-VA	Virginia	3444
			US-VT	Vermont	5458
			US-WA	Washington	16,567
			US-WI	Wisconsin	5129
			US-WV	West Virginia	3322
			US-WY	Wyoming	6161

*Self-rated health* (SRH) is assessed with a single-item measure with five response options. Exact wording and response categories vary between the surveys. SRH has been shown to be a robust predictor of mortality in the context of industrialized countries (Jylhä 2009). The BRFSS measures self-rated health (‘Would you say that in general your health is …’) with five response categories, ‘Poor’, ‘Fair’, ‘Good’, ‘Very good’, and ‘Excellent.’ The EVS asks ‘All in all, how would you describe your state of health these days? Would you say it is …,’ and the response options provided are ‘Very poor’, ‘Poor’, ‘Fair’, ‘Good’, and ‘Very good.’ Similar to Olafsdottir et al. (2013), we opted for a relative concordance interpretation (Jürges et al. 2008) to harmonize the response formats and count European respondents who reported ‘Very poor’ or ‘Poor’ health, as well as American respondents reporting ‘Poor’ or ‘Fair’ health as reporting ‘Poor health’. This left us with poor health prevalence rates of 15.4 and 10.3% in the US and European samples, respectively (Table 2).

**Table 2:** Descriptive statistics by sample, 2008

Variable	Category	US sample (*N* = 269,105)	European sample (*N* = 54,066)
Health	% Poor	15.4	10.3
	% Not poor	84.6	89.8
Education	% Low	5.6	28.5
	% Middle	28.7	39.8
	% High	65.7	31.7
Sex	% Male	38.9	44.5
	% Female	61.1	55.5
Age	Mean	53.1	47.9
	Standard deviation	(12.8)	(14.0)

*Education* is measured based on the ISCED-1997 classification. We distinguish between three key educational groups: Not having completed high school (ISCED 0–2), high school degree (ISCED 3), and further education (ISCED 4–6). We focus on education as a socioeconomic status indicator for several reasons (Präg et al. 2017). First, education reflects both individuals’ material and non-material resources and their social status in a broad fashion. Second, the ISCED, with its high degree of cross-national standardization, allows to meaningfully compare the educational groups across countries. Third, educational attainment is usually completed in early adulthood and remains for the most part stable across the life course, thus reducing the chance of reverse causation: Unlike occupational status, educational degrees do not change even when an adult experiences a health shock. Fourth, compared to income, which usually comes with a large proportion of non-respondents, education is a comparably easy-to-measure indicator of socioeconomic status. Finally, education is a meaningful measure for the socioeconomic status of both men and women and for those outside of the labor force.

We use logistic regression models for each country and US state separately to model the log odds of reporting poor health, adjusted for sex and age. Based on these models, we predicted probabilities of reporting poor health for those with more than and less than high school. We then calculated the difference in adjusted prevalence rates of poor health in the two groups (less than minus more than high school) as a measure of absolute educational inequalities in SRH. Finally, we calculated the ratio of the prevalence rates in the two groups (less than over more than high school), indicating relative educational inequalities in SRH. 95% confidence intervals (CI’s) were obtained by means of the delta method.

## Results

**Fig. 1:**
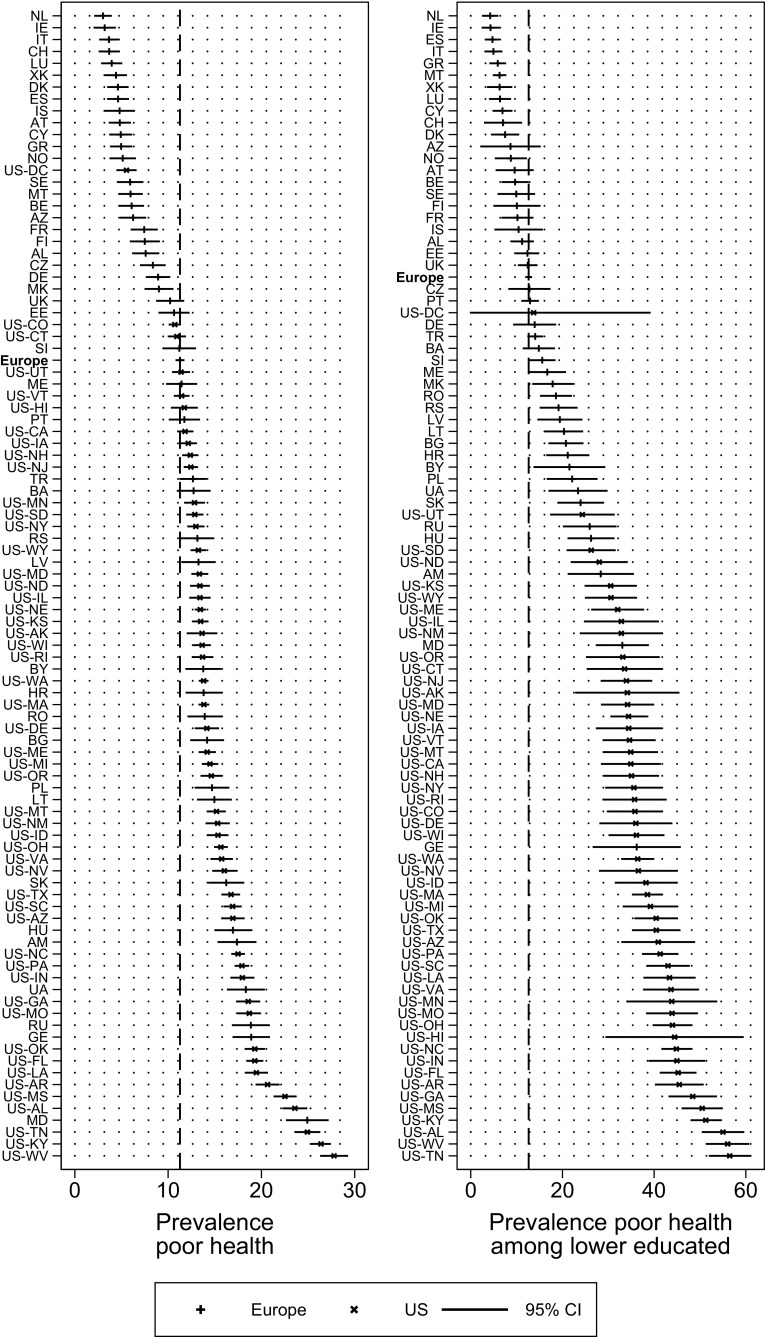
*Left* panel prevalence rate of reporting poor health in European countries and US federal states in 2008; *right* panel prevalence rate of reporting poor health in European countries and US federal states among the lower educated in 2008. Adjusted for age and gender. *Dashed line* denotes population-weighted European Values Study average. 95% CI’s in *right* panel truncated when <0. Abbreviations expanded in Table 1

The left panel of Fig. 1 presents overall prevalence rates of poor SRH in European countries and US federal states. There is substantial variation, ranging from 3.0% reporting poor health in the Netherlands to 27.8% reporting poor health in West Virginia. European countries generally exhibit lower prevalence rates of poor SRH than the US, but this pattern has exceptions. The District of Columbia ranges among the European countries with a relatively low prevalence rate of poor health (5.5%), and a number of Eastern European countries, such as Georgia, Russia, and Ukraine, show prevalence rates in the range of the US states. In 43 US states, the prevalence rates of poor health were significantly above the European (EVS) average (dashed line). The right panel of Fig. 1 shows the prevalence of poor SRH among the lower educated. As expected, the prevalence of poor SRH among the lower educated is substantially higher than in the general population. There are marked differences between the US and Europe, with all states (except Washington, DC) showing a sometimes manifold higher rate than the European average. Again, it is only the Eastern European countries such as Georgia, Moldova, and Armenia that have comparable rates of poor health among their lower educated.

**Fig. 2:**
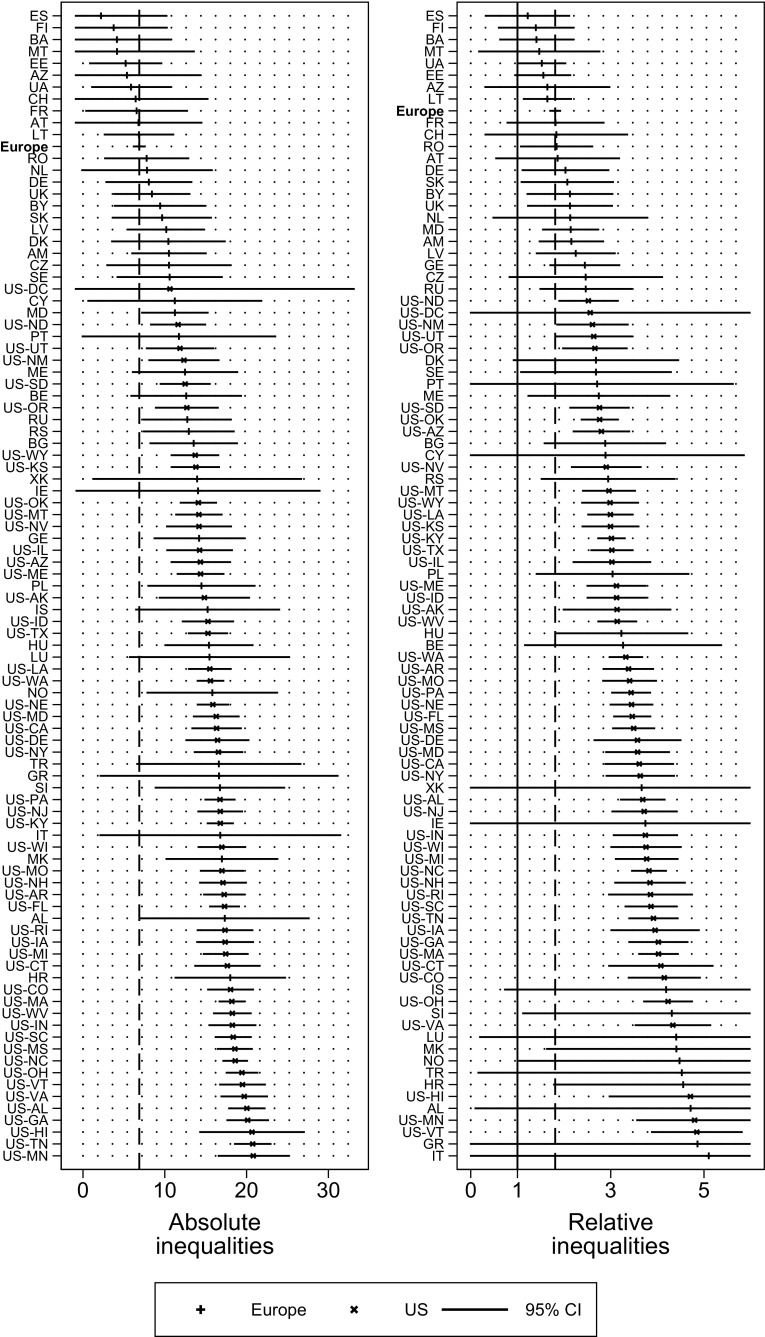
*Left* panel absolute inequalities in reporting poor health (rate difference low educated minus high educated) in European countries and US federal states in 2008; *right* panel relative inequalities in reporting poor health (rate ratio low educated over high educated) in European countries and US federal states in 2008. Adjusted for age and gender. *Dashed line* denotes population-weighted European Values Study average. 95% CI’s in left panel truncated when <−1, 95% CI’s in right panel truncated when <0 or >6. Abbreviations expanded in Table 1

The left panel of Fig. 2 shows the rate differences in poor SRH between lower and higher educated respondents. In North Dakota, for instance, the rate difference is 11.6%, indicating that the prevalence rate of poor SRH among the lower educated is 11.6% points higher than among the higher educated. Poor SRH is generally more prevalent among the lower educated than among the higher educated. Comparing the absolute inequalities shows that differences are usually higher in the US, but a substantial number of European countries exhibit rate differences similar to those found in the US. Croatia, Albania, and Slovenia are the most striking examples for large absolute educational inequalities in SRH in Europe. All US states (except Washington, DC) exhibited greater rate differences than the European average. The right panel shows relative educational inequalities in SRH as expressed by the rate ratio of poor SRH among the lower educated compared to the higher educated. For example, in Lithuania, the rate ratio amounts to 1.6, denoting that the lower educated are 60% more likely to report poor health compared to the higher educated. In a number of countries and states, 95% confidence intervals of the rate ratios include the value 1, indicating that the point estimates are not statistically significant at the conventional level. Among those places where the rate ratio is significantly different from 1, the range is from 1.6 in Lithuania to 4.8 in Vermont. Again, while a large number of European countries exhibit substantially lower rate ratios than the US federal states, many European countries have ratios similar to those of the US, e.g., Croatia (4.6), Norway (4.5), or Macedonia (4.4). The dashed line indicates that all US states except the District of Columbia show greater relative educational inequalities in SRH than Europe.

## Discussion

Our study generated a number of key messages. First, a comparison of the federal states of the US shows substantial heterogeneity in the extent of health inequalities within the US. This is in line with findings of other studies (Chetty et al. 2016; Murray et al. 2006). However, when comparing the within-US variation with the variation within Europe, where smaller health inequalities are more common than in the US, we can see that the potential for reducing health inequalities in the US is likely bigger than what can be inferred from within-US comparisons only. This finding underlines the importance of a cross-nationally comparative perspective for the study of health inequalities.

Second, there is a striking resemblance between some Eastern European countries and large parts of the US in terms of inequalities in self-rated health. While the prevalence of poor self-rated health and educational inequalities in health are generally greater in the US than in Europe, some of the poorest countries of Europe show health inequalities and poor health to the same extent as parts of the US. Our comparison across many rich and poor industrialized countries shows surprising similarities between countries where these would not be expected—for instance, Bulgaria and Belgium for absolute inequalities—and identifying causes for these patterns will be a challenge for future comparative health research (Mackenbach 2012; Präg et al. 2016).

Third, we also show that the poor health prevalence among the lower educated in the US is much higher than in most European countries. Yet, the share of the lower educated among the US population is significantly smaller than in Europe. Thus, only improving the health of the lower educated in the US is unlikely to eradicate the US health disadvantage to other industrialized countries. To close the gap between the US and the other countries, public health policies to improve population health have to also target those with medium levels of education.

Fourth, the patterns found in our study provide important evidence for the debate about the causes of health inequalities. While public welfare provision is generally low across the US states, health inequalities vary substantially in size across states. A possible interpretation for this finding would be that lack of public welfare provision cannot be the only driver of health inequalities. Research has pointed out similarities between the health care systems of the US and those of Eastern Europe. While health care cannot be the only explanation of the US disadvantage in population health, it might, however, be an important factor underlying health inequalities. The US suffers from a lack of universal health care access, and some problems of the health care system in Eastern Europe, like its weakness of primary care, its specialist orientation, and great fragmentation are also found in the US. Furthermore, similarities in the often rudimentary conditions of social policies, both in the US and in Eastern Europe, can provide explanations for the patterns revealed in this study (Avendano and Kawachi 2014).

Fifth, our study points to important directions for future research. Identifying upstream factors which can account for the differences between countries and states will be a major challenge for future research. Income inequality has been suggested to plausibly be a driver of health disparities (Beckfield et al. 2013; Wilkinson and Pickett 2008). Creating a comparable database for comprehensive institutional factors that might serve as explanations not only on the national level, but also on subnational levels, would be an important next step.

Examining heterogeneity within the US only and not within European countries is a shortcoming of our study. Yet, although we only contrasted health inequalities within the US with European countries rather than regions, it became clear that the variability in health inequalities within the US is rather small in comparison to European countries. Still, future research should collect data that allow for more detailed comparisons within Europe. Although the EVS is the largest contemporaneous European micro-dataset in terms of country coverage, the sample sizes within countries prohibit further breakdowns of the data to subnational levels. Attempts to use the pooled European Social Survey for breakdowns by region instead also failed due to the limited sample size.

Given the recent advocacy for global comparisons of health inequalities (Beckfield et al. 2013; Olafsdottir et al. 2013; Präg et al. 2014), our study serves as a reminder to not overlook levels of variation within countries and that understanding local health conditions can give important cues for improving population health.
